# Promoter Methylation of Cancer Stem Cell Surface Markers as an Epigenetic Biomarker for Prognosis of Oral Squamous Cell Carcinoma

**DOI:** 10.3390/ijms232314624

**Published:** 2022-11-23

**Authors:** Yu Kyeong Han, Ha Young Park, Sae-Gwang Park, Jae Joon Hwang, Hae Ryoun Park, Joo Mi Yi

**Affiliations:** 1 Department of Microbiology and Immunology, College of Medicine, Inje University, Busan 47392, Republic of Korea; 2 Department of Pathology, College of Medicine, Inje University, Busan 47392, Republic of Korea; 3 Department of Oral and Maxillofacial Radiology, School of Dentistry, Pusan National University, Yangsan 50612, Republic of Korea; 4 Dental and Life Science Institute & Dental Research Institute, School of Dentistry, Pusan National University, Yangsan 50612, Republic of Korea; 5 Department of Oral Pathology, School of Dentistry, Pusan National University, Yangsan 50612, Republic of Korea

**Keywords:** cancer stem cell surface markers, methylation biomarkers, oral squamous cell carcinoma, promoter DNA methylation, prognosis

## Abstract

Growing evidence suggests that genetic and epigenetic factors, including environmental factors, contribute to the development of oral squamous cell carcinoma (OSCC). Here, we investigated the transcriptional silencing of the *CD24*, *CD44*, *CD133*, and *CD147* genes, which are well-known cancer stem cell surface markers in various cancer types, including OSCC. We first examined the correlation between the transcriptional expression level and reactivation by 5-aza-2′-deoxycytidine (5-aza-dC) and the promoter methylation levels of the four genes in several OSCC cell lines. We observed promoter hypermethylation for the *CD24*, *CD133*, and *CD147* genes at 70%, 75%, and 70%, respectively, in OSCC cell lines compared to normal oral mucosa tissues (<53%), indicating that this methylation pattern is cancer-specific, which was confirmed by bisulfite sequencing analysis. More specifically, the expression and methylation profiles of *CD133* and *CD147* extracted from The Cancer Genome Atlas (TCGA) database were negatively correlated, supporting their epigenetic regulation in primary OSCC tumors. The methylation status of *CD133* and *CD147* was associated with poor survival in patients with OSCC using the TCGA database. Our findings provide additional insight into the abnormal DNA methylation of *CD133* and that *CD147* could be used for the diagnosis and therapeutic treatment of patients with OSCC.

## 1. Introduction

Head and neck squamous cell carcinoma (HNSCC) is one of the most fatal cancers worldwide, with oral squamous cell carcinoma (OSCC) being the most common subtype. OSCC is characterized by high rates of metastasis, recurrence, and resistance to traditional chemotherapy, with more than 350,000 cases and 170,000 deaths from OSCC per year [1,2]. Consequently, the vast majority of patients with OSCC have a poor prognosis. Despite advances in the molecular and genetic mechanisms driving OSCC malignancy, the 5-year-survival rate has not significantly improved [3,4].

An aberrant DNA methylation pattern is a known hallmark of cancer cells [5], with DNA methylation abnormalities associated with tumor suppressor gene (TSG) silencing, leading to cancer progression. Particularly, hypermethylation in cancer cells is frequently observed in transcriptional regulatory elements such as the promoters and enhancers of genes including TSGs [6]. Recent studies using genome-wide methylation analysis have discovered hypermethylation in the promoter regions of TSGs, suggesting that unknown genes regulated by promoter hypermethylation may function to drive cancer development, including TSGs [7]. 

Previous studies have demonstrated the existence of cancer stem cells (CSCs) in multiple solid tumors, including OSCC. Numerous cell surface markers have proved useful for the isolation of subpopulations enriched for CSCs, including CD133, CD44, CD24, and epithelial cell adhesion molecule (EpCAM) [8]. CSCs have recently been isolated from the same organ using markers specific for normal stem cells. Thus, common cell surface markers, particularly CD133 and CD44, can be used to fractionate CSCs in diverse solid tumors [8]. It remains unknown whether these markers represent surrogate markers or if they play a role in regulating CSC function in cancer. We previously showed that *CD133* gene silencing is associated with the hypermethylation of CpG islands in its promoter region, even though CD133 is a known CSC surface marker [9]. Moreover, it is demethylated by 5-aza-dC in tumors, suggesting that additional insights into the dynamics of abnormal DNA methylation and its correlation with gene silencing in human tumors are required. 

CSCs are self-renewable cell types identified in most liquid and solid cancers and contribute to tumor onset, expansion, resistance, recurrence, and metastasis after therapy. The development of therapeutic strategies targeting CSCs relies mainly on the use of cell surface markers to identify, enrich, and/or isolate CSCs. To date, putative OSCC CSCs are being explored, providing evidence for their potential role as novel diagnostic, prognostic, or therapeutic targets. Moreover, several known surface markers of colorectal, glioblastoma, or breast cancer, including CD44, CD133, and CD147, have also been identified in OSCC CSCs. 

CD44, a non-kinase surface transmembrane glycoprotein, has been widely implicated as a CSC marker in diverse solid tumors [8]. Cells overexpressing CD44 possess several CSC traits, such as self-renewal and epithelial-mesenchymal transition (EMT) capability, as well as resistance to chemotherapy and radiotherapy. CD44 is a polymorphic integral membrane glycoprotein that is expressed in a variety of normal and abnormal cells. Several studies have reported that the hypermethylation of the CpG island located in the promoter of *CD44* is associated with transcriptional inactivation, resulting in silencing or decreasing the expression of *CD44* in the prostate [10,11]. CD44^+^CD24^−/low^ is a well-known surface marker of breast cancer stem cell subpopulations [12]. CD133 (also known as *Prominin-1*, *PROM1*) is a highly glycosylated transmembrane protein that defines a broad population of stem cells, including hematopoietic stem cells and endothelial progenitor cells [13].

CD147 (also known as *Basigin*, *BSG*) is widely distributed in several human cells, with high expression in some solid tumors, including OSCC. Therefore, its overexpression is associated with malignancy and poor prognosis in several tumor types involved in cancer cell growth, invasion, and metastasis [14]. Recently, it has also been identified as a CSC marker of head and neck cancer and OSCC [15]. 

In this study, we investigated whether other common CSC surface markers such as *CD44*, *CD133*, *CD147*, and *CD24* could be regulated at the transcriptional level by DNA methylation in OSCC. There is little information on the regulation of these CSC markers by epigenetic mechanisms in OSCC cells. Here, we investigated the epigenetic regulation of the CSC markers CD133, CD147, and CD24 in OSCC cell lines to understand their possible roles in the pathogenesis of OSCC.

## 2. Results

### 2.1. Transcriptional Expression of CSC Surface Markers Is Regulated by Promoter DNA Hypermethylation in OSCC Cell Lines

**Figure 1:**
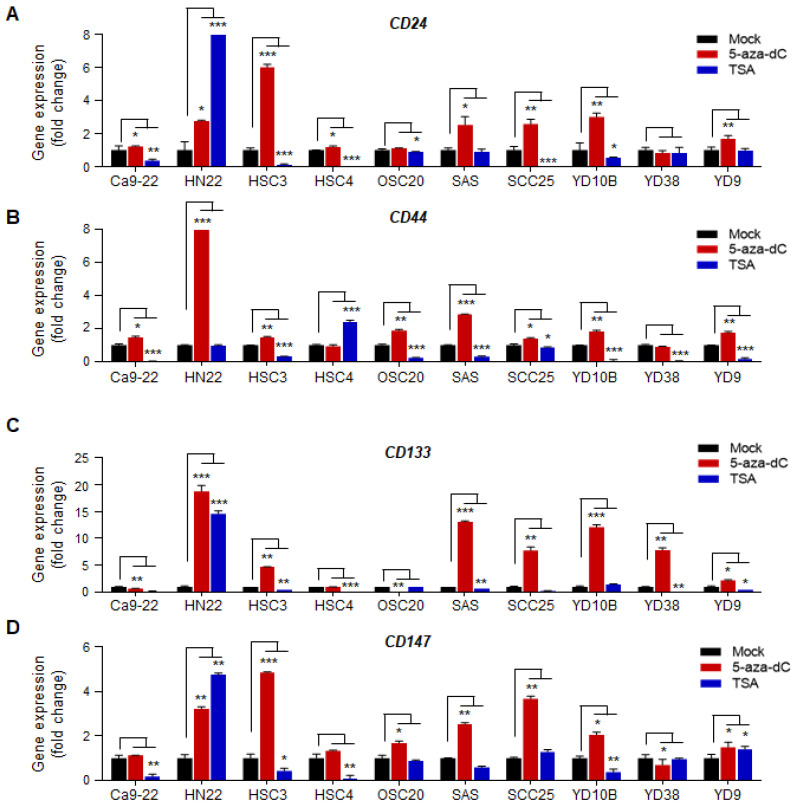
Epigenetic inactivation of CSC surface markers (*CD24*, *CD44*, *CD133*, and *CD147*) in OSCC cell lines. Quantitative real-time RT-PCR analysis was carried out to assess the transcriptional expression level of (**A**) *CD24*, (**B**) *CD44*, (**C**) *CD133*, and (**D**) *CD147* genes in OSCC cell lines before and after treatment with 5-aza-dC (5 μM) for 72 h and TSA (0.3 μM) for 18 h. The expression levels of the genes were internally normalized to the expression levels of *GAPDH*, and the normalized expression for each gene before 5-aza-dC treatments was set to one. The asterisks indicate significant increases in gene expression after 5-aza-dC treatment (*p* < 0.05). All data were statistically analyzed using the Student’s *t*-test. * *p* < 0.05; ** *p* < 0.01; *** *p* < 0.001; no asterisk, not significant. A two-tailed Student’s *t*-test was used to compare data between the two groups.

To investigate whether the transcriptional expression of CSC surface markers (*CD24*, *CD44*, *CD133*, and *CD147*) is regulated by epigenetic mechanisms in oral squamous cell carcinoma cells, we first treated ten OSCC cell lines (Ca9-22, HN22, HSC3, HSC4, OSC20, SAS, SCC25, YD10B, YD38, and YD9) with 5-aza-2′-deoxycytidine (5-aza-dC, DAC) and determined whether these genes were reactivated after 5-aza-dC treatment by real-time RT-PCR. In addition, we treated OSCC cell lines with trichostatin A (TSA) to test whether these genes are regulated by histone modifications. We also verified the transcriptional expression of these genes in the OSCC lines using real-time RT-PCR. Treatment with 5-aza-dC increased the transcriptional expression of all four genes in different OSCC cell lines. *CD147* and *CD24* were also significantly re-expressed by 5-aza-dC treatment in five out of ten OSCC cell lines (HN22, HSC3, SAS, SCC25, and YD10B) (Figure 1A,D). CD44 was significantly re-expressed in three cell lines (HN22, OSC20, and SAS) (Figure 1B). In addition, CD133 was significantly re-expressed by 5-aza-dC treatment in six OSCC cell lines (HN22, HSC3, SAS, SCC25, YD10B, and YD38) (Figure 1C). Thus, we next investigated the correlation between the transcriptional expression and the promoter DNA methylation of these genes. 

### 2.2. Correlation between the Transcriptional Expression of CD133 and CD147 and Promoter Hypermethylation in OSCC Cell Lines

**Figure 2:**
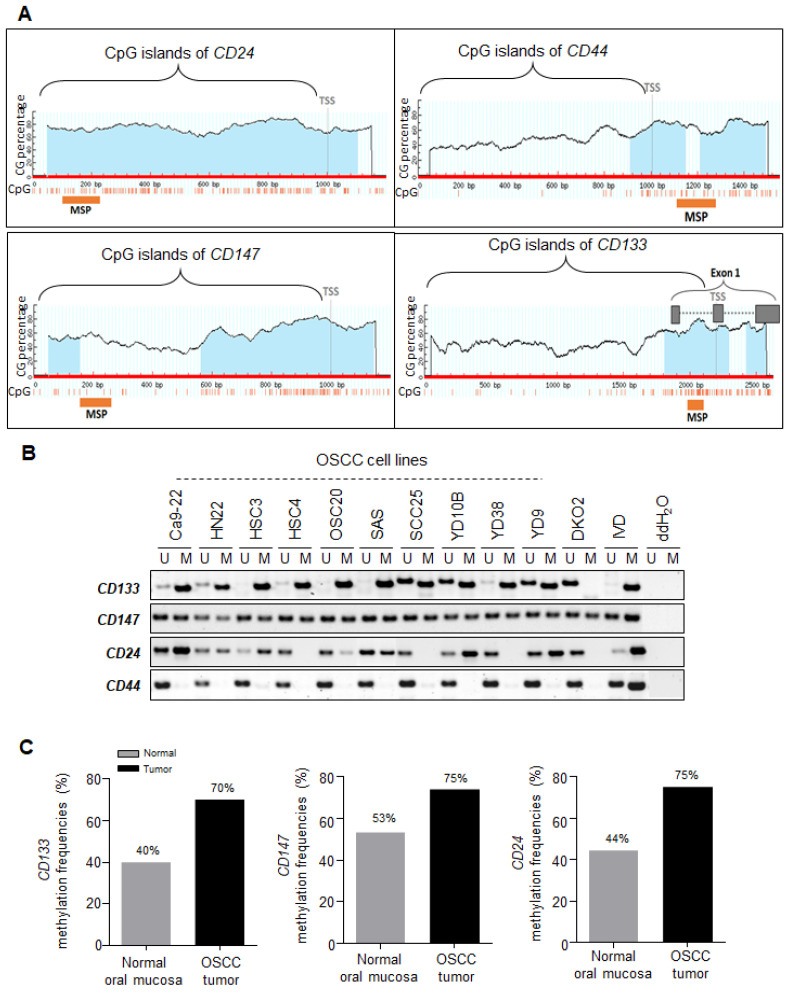
Promoter DNA methylation analysis of the *CD24*, *CD44*, *CD133*, and *CD147* genes in OSCC cell lines, primary OSCC tumor tissues, and normal oral mucosal tissues. (**A**) Schematic structure of the CpG islands at the promoter regions for *CD24*, *CD44*, *CD133*, and *CD147.* (**A**) The primer locations for MSP were indicated as an orange bar for each gene. (**B**) MSP results in OSCC cell lines. Gel pictures describe the methylation analysis by MSP. DNA methyltransferase 1 and 3b knockout HCT116 cells (DKO) were included as positive controls. The PCR products recognized the unmethylated (U) and methylated (M) genes. DKO cells were used for the unmethylated control. IVD = in vitro methylated control; ddH_2_O = water control containing no DNA. (**C**) Methylation frequency of the *CD24*, *CD133*, and *CD147* genes between the samples from patients with OSCC (*n* = 102) and the normal oral mucosal tissue samples (*n* = 45).

Transcriptional reactivation was observed in these genes after 5-aza-dC treatment in OSCC cell lines, indicating that the reactivation observed was due to DNA demethylation in their promoter region. To determine the correlation between transcriptional gene silencing and promoter DNA methylation, we verified whether these genes had typical CpG islands in their promoter regions from the UCSC database (Figure 2A). Figure 2A shows that the *CD24*, *CD44*, *CD133*, and *CD147* genes have typical CpG islands in their promoter regions, indicating that these genes might be regulated by promoter DNA methylation. Therefore, we queried the promoter sequences of these genes and designed primers for their methylation analysis. 

Next, we assessed the DNA methylation patterns of *CD24*, *CD44*, *CD133*, *and CD147* in ten OSCC cell lines (Ca9-22, HN22, HSC3, HSC4, OSC20, SAS, SCC25, YD10B, YD38, and YD9) using methylation-specific PCR (MSP) analysis (Figure 2B). First, we observed no methylation of CD44 in any of the OSCC cell lines tested, suggesting that the transcriptional gene expression of CD44 is not correlated with promoter DNA methylation. However, *CD133* showed either complete (Ca9-22, HN22, HSC3, HSC4, OSC20, and YD38) or partial hypermethylation (SAS, SCC25, YD10B, and YD9) in all OSCC cell lines, while *CD147* showed partial hypermethylation in all OSCC cell lines. The *CD24* gene was mostly hypermethylated in OSCC lines, except for the HSC4, SCC25, and YD38 cells. Overall, *CD24*, *CD147*, and *CD133* gene expression at the transcriptional level was associated with their promoter DNA methylation in OSCC cell lines, except for the *CD44* gene. Based on these data, we eliminated *CD44* from further experimental analyses because *CD44* is not methylated in OSCC cell lines.

### 2.3. Promoter Hypermethylation of CSC Surface Markers Frequently Occurred in Primary OSCC Tumor Tissues

Having confirmed that the promoter hypermethylation of CSC surface markers regulates their transcriptional expression in OSCC lines, we next investigated the methylation status of these genes in primary OSCC tumors. We first established that the promoter methylation of CD44, CD24, CD147, and CD133 was absent in normal oral mucosal tissues (*n* = 45) (Supplementary Figure S1), indicating that the methylation of these genes may represent an abnormal event in tumor cells. To test the methylation frequencies of CSC surface markers in primary OSCC tumors, we expanded our methylation analysis to include a large series of primary OSCC tumor samples (*n* = 102, Table 1). We observed a significantly increased methylation frequency in the primary OSCC samples (Figure 2C). The methylation of *CD133*, *CD147*, and *CD24* was observed in 70%, 75%, and 75% of the primary OSCC tumors, respectively, confirming a cancer-specific methylation pattern of these CSC surface markers. 

**Figure 3:**
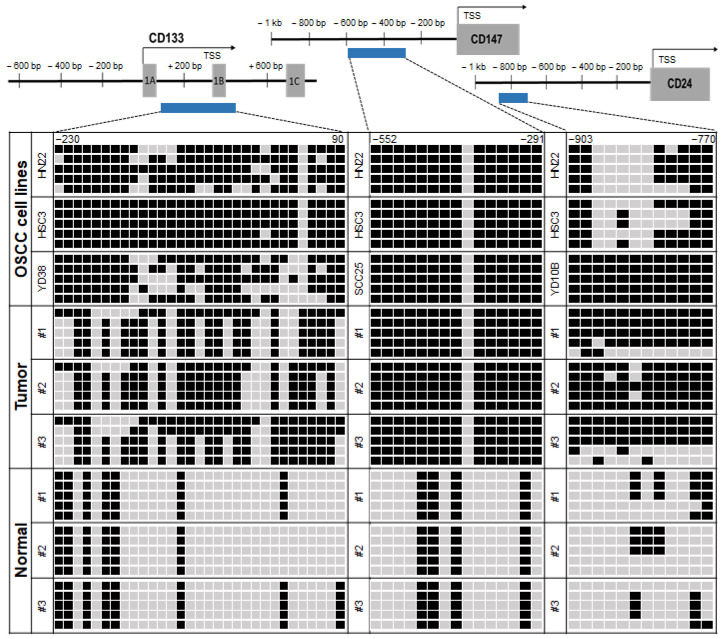
Representative bisulfite sequencing results of the CpG islands in the *CD24*, *CD133*, and *CD147* gene promoter regions in OSCC cell lines, primary OSCC tumors (*n* = 3), and normal oral mucosal tissues (*n* = 3). The locations of the CpG sites (*CD24*: upstream region from −903 to −770; CD133: exon 1 region from −230 to +90 [9]; and CD147: upstream region from −552 to −291) relative to the transcription start sites (TSSs) of exon 1 are shown. Each box represents a CpG dinucleotide. The black boxes represent methylated cytosines, and the white boxes represent unmethylated cytosines.

We next performed bisulfite genomic sequencing analysis to confirm the DNA methylation status of *CD133*, *CD147*, and *CD24* at the genomic level in representative OSCC lines. The bisulfite sequencing results showed that *CD133*, *CD147*, and *CD24* were densely methylated in the HN22, HSC3, YD38, SCC25, and YD10B cells (Figure 2B and Figure 3). In the HN22 and HSC3 cells, we confirmed significantly increased methylation in all genes (51.7–53.3% methylated CpG sites in *CD24*, 72.9–96.1% CpG methylated sites in *CD133*, and 93.3% methylated CpG sites in *CD147*). The YD38, SCC25, and YD10B cells showed methylated sites for *CD133* (72.9%), *CD147* (93.3%), and *CD24* (100%), respectively. We also analyzed the bisulfite genomic sequencing results of the representative primary OSCC tumor samples (*n* = 3) and normal oral mucosal tissue samples (*n* = 3), and we observed that the primary OSCC tumor samples showed highly dense CpG methylated sites in *CD133* (61.3–70.3%), *CD147* (93.3%), and *CD24* (66.7–93.3%). Contrary to their methylation level in tumor tissues, these genes showed very low CpG methylated sites in *CD133* (19.4–25.8%), *CD147* (26.7%), and *CD24* (5–25%) in the normal oral mucosal tissue samples. Our data suggest that the robust cancer-specific hypermethylation in the promoter regions of *CD133*, *CD147*, and *CD24* by bisulfite genomic sequencing analysis is in direct agreement with our MSP results in OSCC cell lines (Figure 3). Interestingly, *CD147* gene methylation was also consistent with the MSP results, even though the bisulfite sequencing region was not included within the MSP region. 

### 2.4. Correlation between DNA Methylation and the Transcriptional Expression of CD133 and CD147 Using the TCGA Database

**Figure 4:**
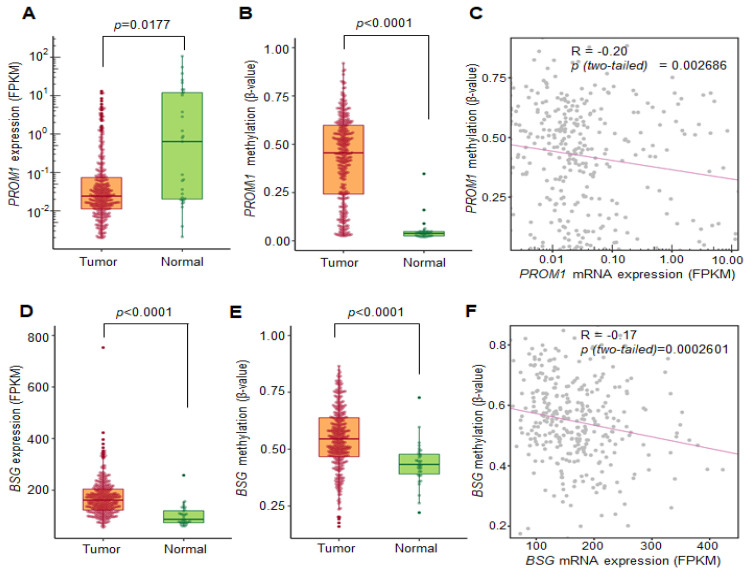
Correlation analysis between gene expression level and promoter methylation level from the TCGA database. (**A**,**D**) The expression levels of *CD133 (PROM1)* and *CD147 (BSG)* in tumor tissues were inversely correlated with the promoter DNA methylation rates. Expression levels of (**A**) *CD133* and (**D**) *CD147* in primary OSCC tumors (*n* = 328) compared to normal oral mucosa (*n* = 32), extracted from the TCGA HNSCC database. (**B**,**E**) The methylation levels of the CpG sites of (**B**) *CD133* and (**E**) *CD147* in primary OSCC tumors (*n* = 338) compared to normal oral mucosa (*n* = 34), extracted from the HNSCC TCGA database. (**C**,**F**) Negative correlation between the gene expression and promoter methylation of (**C**,**F**). Spearman’s correlation analysis was performed between the methylation (vertical axis) and gene expression (horizontal axis) of the *CD133* (*R =* −0.20, *p =* 0.002686) and *CD147* genes (*R =* −0.17, *p =* 0.0002601). The Spearman’s correlation coefficients and *p*-values are shown in each plot.

To confirm the correlation between gene expression and DNA methylation for the *CD133* and *CD147* genes in a large cohort of patients with OSCC, we analyzed the profiles of the DNA methylation and transcriptional expression data of patients with HNSCC from the TCGA database [16]. Although *CD24* satisfied our criteria for cancer-specific methylation with high-frequency methylation in cell lines, undetectable methylation in normal oral mucosa, and frequent methylation in primary OSCC tumor samples, we decided to eliminate *CD24* from further experimental analyses because the information on the CpG probes for *CD24* was not available for genome-wide methylation profiles from the TCGA HNSCC database. First, we plotted the FPKM expression values of *CD133* and *CD147* against the average β value for the genes expressed in the TCGA dataset. The box plot shows that the *CD133* (*PROM1*) (Figure 4A,B) and *CD147* (*BSG*) (Figure 4D,E) genes were significantly hypermethylated in tumors compared to normal tissues; inversely, their mRNA expression levels were downregulated in tumor tissues. The scatter plots between mRNA expression and the DNA methylation profiles indicated that both *CD133* and *CD147* expression were inversely correlated with DNA methylation in the TCGA dataset (R = −0.20, *p* = 0.002686 for *CD133* and R = −0.17, *p* = 0.0002601 for *CD147*, respectively) (Figure 4C,F). These data suggest that the methylation of both *CD133* and *CD147* shows a significant negative correlation with gene expression in the TCGA database, which is consistent with our experimental results (Figure 1 and Figure 2), and it further suggests their important roles as methylation biomarkers in OSCC tumors. 

### 2.5. CD147 and CD133 Protein Expression Is Downregulated in Primary OSCC Tumor Tissues 

**Figure 5:**
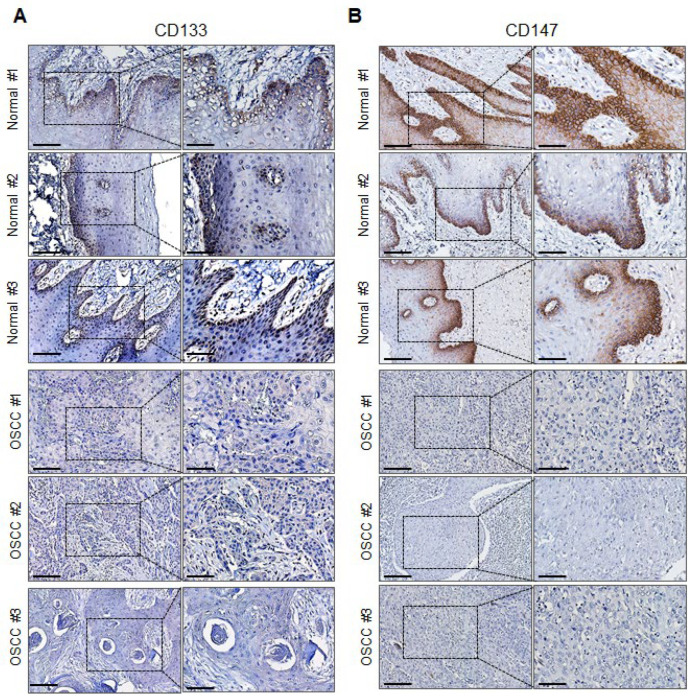
Protein expression levels of CD133 and CD147 in primary OSCC tumors and normal oral mucosal tissue samples. The representative immunohistochemical analysis results show (**A**) CD133 and (**B**) CD147 expression in primary OSCC tissues and normal oral mucosal tissues (left images: × 40, scale bar, 50 µm; right images: × 20, scale bar, 100 µm).

To further explore the downregulation of CD133 and CD147 genes during OSCC tumor development, we examined the protein expression of both genes in primary OSCC and normal oral mucosal tissues by immunohistochemical (IHC) staining. The normal oral mucosal tissues indicated mostly cytoplasmic or nuclear staining for CD133, but the cytoplasmic or membranous staining for CD147 (Figure 5) did not. Furthermore, the protein expression of CD133 was detected in both the basal and parabasal cells, whereas CD147 was detected mostly in the basal cells of the normal oral mucosal tissues. Overall, the normal oral mucosal tissues showed strong CD147 and CD133 protein expression (Figure 5). In contrast, these proteins were downregulated in both the nucleus and cytoplasm of the primary OSCC tissues.

### 2.6. Promoter DNA Hypermethylation of CD133 and CD147 Is Associated with Poor Prognosis in OSCC Patients

**Figure 6:**
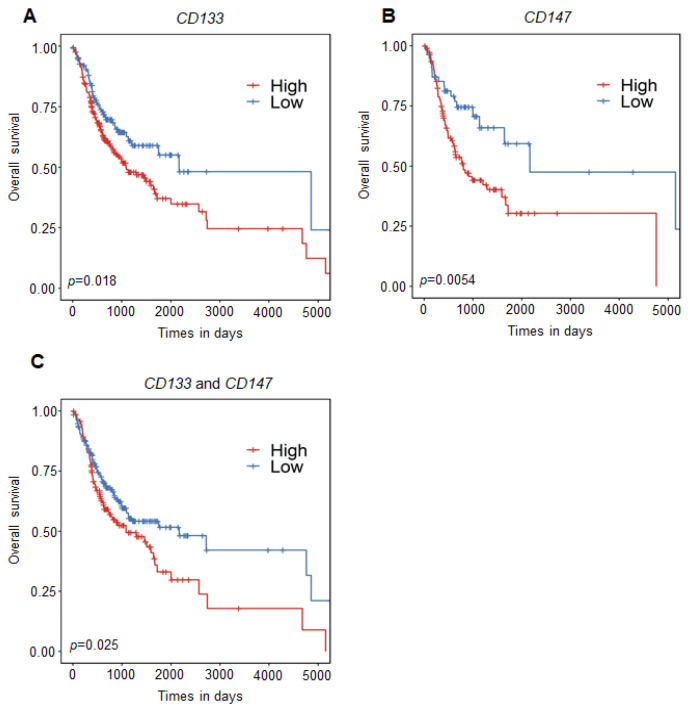
Kaplan-Meier curves showing the effect of CD133 and CD147 DNA methylation on overall survival among patients with OSCC (*n* = 338) from the HNSCC TCGA dataset. The overall survival for (**A**) CD133 (*p* = 0.018) and (**B**) CD147 (*p* = 0.0054), respectively, is shown. A probability of <0.05 was considered to represent a statistically significant difference. (**C**) Combination of CD133 and CD147 gene methylation (*p* = 0.025). The clinical information of patients with OSCC (*n* = 338) was divided into two groups according to their methylation status (β-values) on CD133 (methylation high (red line), *n* = 209 and methylation low (blue line), *n* = 129), CD147 (methylation high (red line), *n* = 113 and methylation low (blue line), *n* = 55), and a combination of CD133 and CD147 (methylation high (red line), *n* = 143 and methylation low (blue line), *n* = 195).

To explore whether the methylation status of both *CD133* and *CD147*, as potential biomarkers, have an impact on the overall survival of patients with OSCC, we subtracted the patients with OSCC from the TCGA HNSCC cohort and well-annotated clinical data that could be correlated with survival and gene methylation status. DNA methylation data (CpG sites) with clinical information (*n* = 338) from the TCGA database was divided into two groups (high- and low-methylation groups) according to their methylation status in *CD133* or *CD147*. Kaplan–Meier analyses were performed for each CpG in the promoter region or combinations of CpGs. We observed a significantly increased risk of mortality when either *CD133* or *CD147* were methylated in patients with OSCC. The Kaplan–Meier survival curves for *CD133* (Figure 6A) and *CD147* (Figure 6B), individually, showed that the DNA methylation of each was significantly associated with decreased survival (*p* = 0.0054 for *CD133* and *p* = 0.018 for *CD147*). These results support our hypothesis that the methylation of *CD133* and *CD147* may play an important role as a prognostic biomarker in clinical applications. We investigated whether the combined methylation of the two genes could have an impact on overall survival in this cohort, and we observed a significantly worsened survival rate with the methylation of both genes (*CD133* and *CD147*) (Figure 6C, *p* = 0.025). Taken together, these data support our hypothesis that the DNA methylation of CSC surface markers (*CD133* and *CD147*) may play an important role in OSCC development and serve as a prognostic biomarker for patients with OSCC. 

## 3. Discussion

Epigenetic alterations, including DNA methylation and histone modifications, play well-established roles in the development and progression of different types of cancers [17]. The DNA methylation of promoter CpG islands is strongly associated with gene silencing and is known to be a frequent cause of the loss of expression of TSGs and other genes involved in tumor formation [18]. The development of genome-wide analysis technology has enabled the identification of several other candidate TSGs associated with gene silencing in different types of cancers [19]. 

We previously identified that *CD133*, which is a marker of colon and brain cancer stem cells, is transcriptionally regulated by the promoter DNA methylation of CpG islands in colorectal cancer [9]. Moreover, the gene expression of other CSC surface markers such as *CD44*, *EpCaM*, and *CD34* was also found to be regulated by promoter DNA hypermethylation in colorectal cancer [20]. However, CSC surface markers in OSCC remain under-explored. In this study, we tested the epigenetic regulation of several CSC surface markers (CD24, CD44, CD133, and CD147) that have been implicated as CSC surface markers in OSCC [21,22]. Our results demonstrated that the hypermethylation of CSC surface markers (*CD24*, *CD133*, and *CD147*) significantly downregulated the expression of these genes in both OSCC cell lines and primary OSCC tumors. Moreover, we observed that the hypermethylation of *CD133* and *CD147* was significantly associated with a poor prognosis in patients with OSCC, according to the TCGA database. 

*CD133* has been identified as a CSC surface marker for cancer-initiating cells in several solid malignancies [23,24]. Several groups have reported that *CD133* transcriptional regulation in colon cancer is strongly associated with the DNA hypermethylation of CpG islands in its promoter region. *CD133* is re-expressed via demethylation with 5-aza-dC in colon cancer cells, suggesting that DNA methylation is an important mechanism for *CD133* transcriptional inactivation in colon cancer [9,25]. Our data provide additional evidence that the transcriptional inactivation of *CD133* is regulated by promoter DNA hypermethylation in other cancer types such as OSCC. 

*CD147* is a CSC marker of head and neck cancer but is also involved in the pathogenesis of oral cancer [15]. *CD147* is an important marker for independent growth, angiogenesis, drug resistance, hypoxic survival, invasion, properties attributed to CSCs, and essential molecular events in carcinogenesis [26]. Additionally, CD147 is a marker of undifferentiated human embryonic stem cells [27] and has been reported as a marker of oral CSCs [28]. Recent studies have implicated *CD147* and *CD44* in the modulation of the Wnt/β-catenin pathway [29] in metastatic prostate cancer, a property that is being investigated as a potential therapeutic strategy [22]. It has been reported that a combination of CD44- and CD147-positive cells might be more similar to a CSC population than CD133-positive cells in HNSCC and oral cancer. However, we report here that the transcriptional silencing of *CD44* is not associated with promoter hypermethylation in OSCC (Figure 2). To the best of our knowledge, we are reporting for the first time that the transcriptional silencing of the *CD147* gene is regulated by promoter DNA hypermethylation, and this phenomenon is associated with poor prognosis in patients with OSCC. 

The Cancer Genome Atlas, a landmark cancer genomics program, molecularly characterized over 20,000 primary cancers and matched normal samples spanning 33 cancer types. The TCGA program has contributed to the accumulating genomic, epigenomic, transcriptomic, and proteomic data, and it remains a publicly available resource. These data have led to improvements in the diagnosis, treatment, and prevention of cancer. The TCGA-HNSCC data collection is a part of a larger effort to build a research consortium focused on connecting cancer phenotypes to genotypes by providing clinical information matched to subjects from the TCGA. 

Several studies have reported that CD44^+^/CD24^−^ cells have CSC properties in breast and prostate cancers [12,30]. However, very few reports have demonstrated that CD44^+^/CD24^−^ may be the CSC phenotype in OSCC [31]. We also report, for the first time, that the promoter hypermethylation of *CD24* frequently occurs in OSCC cell lines. We asked whether the methylation status of *CD24* is associated with patients with OSCC from TCGA dataset; unfortunately, the present study had a limitation because there are no available CpG site probes for *CD24* in TCGA methylation profiles, and so we could not analyze the correlation between gene expression and methylation, as well as the survival curve of *CD24* methylation status. However, we analyzed the expression level of *CD24* in normal oral mucosal tissues and primary OSCC tumor tissues from the TCGA dataset. Interestingly, the transcriptional expression of *CD24* was significantly downregulated in primary OSCC tumor samples compared to that of normal oral mucosal tissues (Supplementary Figure S2A). Moreover, by KM analysis, we observed that the lower expression of *CD24* is associated with poor prognosis in patients with OSCC (Supplementary Figure S2B), leading us to assume that primary OSCC tumors with high methylation levels might be associated with poor prognosis in patients with OSCC. 

## 4. Conclusions

In summary, this study identified the epigenetic regulation of CSC surface markers (*CD24*, *CD44*, *CD133*, and *CD147*) in OSCC cell lines, a small cohort of primary OSCC tumors, and normal oral mucosal tissues. Importantly, we identified that the *CD24*, *CD133*, and *CD147* genes are regulated by promoter DNA hypermethylation in a cancer-specific manner in OSCC cell lines, and we validated this phenomenon in primary OSCC tumors with frequent hypermethylation. The methylation levels in the promoter regions of these genes were also confirmed by bisulfite genomic sequencing analyses, suggesting that methylation levels are significantly increased in OSCC cell lines and primary tumors compared to those of normal oral mucosa tissues. Using the TCGA database, we confirmed a significant negative correlation of *CD133* and *CD147* between methylation and expression levels, suggesting their biological roles during the development of OSCC. Moreover, we showed that the methylation levels of *CD133* and *CD147* are associated with poor prognoses in patients with OSCC in an expanded methylation dataset of patients with OSCC from the TCGA database. Our work demonstrated that the promoter hypermethylation of *CD133* and *CD147* frequently occurs in primary OSCC tumors and patients with OSCC from the TCGA database, suggesting that epigenetic changes might explain an important mechanism regulating CSC populations. Moreover, the methylation of *CD133* and *CD147* may be a potential biomarker for prognosis in patients with OSCC, providing a new integrative approach for the screening and diagnosis of patients with OSCC. Based on our study, in terms of epigenetic changes by the promoter DNA methylation of CSC surface markers in multiple cancers, this might contribute to the development of a new therapeutic approach to regulate CSC population by epigenetic mechanisms. 

## 5. Materials and Methods

### 5.1. Cell Culture and Treatment 

Ten human OSCC cell lines (Ca9-22, HSC4, OSC20, SAS, SCC25, HN22, YD10B, YD38, YD9, and HSC3) were used. The Ca9-22 and HSC4 cell lines were cultured in MEM/EBSS medium (HyClone, Logan, UT, USA). The OSC20, SAS, and SCC25 cell lines were cultured in a 1:1 mixture of Dulbecco’s modified Eagle’s medium and Ham’s F-12 Nutrient Mixture (DMEM/F12; HyClone). The HN22, YD10B, YD38, YD9, and HSC3 cell lines were cultured in a Dulbecco’s modified Eagle’s medium. All cell culture media were supplemented with 10% fetal bovine serum (HyClone, Logan, UT, USA) and 1% antibiotic-antimycotic solution (Gibco, Grand Island, NE, USA). All cell lines were incubated at 37 °C under 20% O_2_ and 5% CO_2_. To investigate the effects of 5-aza-dC, cells were treated with 5 μM 5-aza-dC (Sigma, St. Louis, MO, USA) for 72 h.

### 5.2. Primer Design

For expression studies using RT-PCR, we designed primers using the open-access program Primer3. Primer sequences for MSP analysis were designed using MSPPrimer, and their locations in the *CD24*, *CD44*, *CD147*, *and CD133* promoters are shown in Figure 2. All primer sequences are listed in Supplementary Table S1. 

### 5.3. Quantitative Real-Time RT-PCR

Total RNA was extracted from cell lines using the RNeasy Mini Kit (Qiagen, Hilden, Germany), treated with Dnase (Qiagen, Hilden, Germany), and 1 μg of total RNA was converted using the Superscript II First Strand cDNA Synthesis Kit (Invitrogen, Waltham, USA) according to the manufacturer’s instructions. The primers used for the expression studies are listed in Supplementary Table S1. Quantitative RT-PCR was performed on a CFX96^TM^ Real-Time PCR Detection System (Bio-Rad, Hercules, CA, USA) using SYBR Green Master Mix (Thermo Scientific, Waltham, MA, USA). The expression levels of the target genes were normalized to *GAPDH* levels, and the relative quantification of expression was calculated using the 2^−ΔΔCt^ method. 

### 5.4. Methylation Analyses

For methylation-specific PCR (MSP) analysis, DNA was extracted using the standard phenol-chloroform extraction method. The bisulfite modification of genomic DNA was performed using the EZ DNA methylation kit (Zymo Research, Irvine, CA, USA). We performed the methylation analysis of the *CD24*, *CD44*, *CD133*, and *CD147* promoters using MSP primer pairs covering the putative transcriptional start site in the 5′ CpG island with 1 μL of bisulfite-treated DNA template and JumpStart Red Taq DNA Polymerase (Sigma, St. Louis, MO, USA) for amplification, as previously described [32]. 

### 5.5. Bisulfite Sequencing Analysis

Genomic DNA (1 μL) from each sample was bisulfite-converted using an EZ DNA methylation kit (Zymo Research, Irvine, CA, USA) following the manufacturer’s protocol. The PCR conditions and primer sequences are listed in Supplementary Table S1. The PCR amplicons were gel-purified and subcloned into the pCRII-TOPO vector (Invitrogen, Waltham, MA, USA). At least ten clones were randomly selected and sequenced on an ABI3730xl DNA analyzer to ascertain the methylation pattern of each locus. 

### 5.6. Tissue Samples

Paraffin-embedded tissue specimens were retrieved from patients with OSCC who were diagnosed and surgically treated at the Department of Oral and Maxillofacial Surgery, Pusan National University Dental Hospital, between 2009 and 2014. For normal oral mucosal tissues (*n* = 45), fresh healthy tissue that had been obtained during dental procedures, such as gingivoplasty, third molar extraction, or implant surgery, was used. Clinicopathological features, including age, sex, histopathological grade, and TNM stage, are summarized in Table 1. This study was approved by the Institutional Review Board (IRB) of Pusan National University Dental Hospital (IRB No. PNUDH-2019-046), and written informed consent was obtained from all patients.

### 5.7. Validation Analysis from the TCGA Database

The head and neck squamous cell carcinoma (HNSC) transcriptome profiling dataset (RNA-Seq; *n* = 546) and DNA methylation dataset (HumanMethylation 450k Illumina; *n* = 580) from TCGA were downloaded from the GDC Data Portal website. Samples from the tonsil, larynx, oropharynx, hypopharynx, and lip were excluded based on anatomical subdivision. To compare the expression levels (FPKM) between the tumor and normal tissues, 328 primary tumors and 32 normal samples were used. To compare the DNA methylation levels (β-value), 338 tumor and 34 normal tissue samples were used. The survival differences between the groups with high and low methylation levels and 338 patients with tumors were used. To choose the best β value cut-off for grouping the patients most significantly, all β values were examined, and the value yielding the lowest log-rank *p* value was selected. Statistical analyses and visualization were performed using R version 3.6.3. Spearman’s correlation analysis was performed between the methylation and gene expression of the two genes (*CD133* and *CD147*). 

### 5.8. Immunohistochemistry (IHC)

Serial 4 μm sections were applied to 3-aminopropyltriethoxysialne-coated slides (Sigma), deparaffinized, and rehydrated in xylene and serially diluted ethanol. Endogenous peroxidase was blocked by incubation in a 3% aqueous hydrogen peroxide solution, after which heat-induced antigen retrieval was performed. Primary antibodies against CD133 (1:100; #66666-1) (Proteintech, Rosemont, IL, USA) and CD147 (1:200; #13287) (Cell Signaling Technology, Danvers, MA, USA) with a benchmark autostainer (Roche Tissue Diagnostics, Oro Valley, AZ, USA) were used following the manufacturer’s protocol. The primary antibody was incubated at RT for 32 min, after which the sections were labeled with an automated immunostaining system using an I-View detection kit (Roche Tissue Diagnostics, Oro Valley, AZ, USA). The immunostained sections were lightly counterstained with hematoxylin, dehydrated in ethanol, and cleared in xylene.

### 5.9. Statistical Analysis 

All experimental data are reported as means ± standard deviations, and all experiments were repeated three times. Statistical significance was set at *p* < 0.05. GraphPad Prism 9.0 software was used for the statistical analysis. Spearman’s correlation analysis was performed between methylation and gene expression from the TCGA database. Statistical analyses and visualization from the TCGA database were performed using R version 3.6.3. 

## Figures and Tables

**Table 1:** Characteristics of patients with OSCC in this study.

Characteristics			N
Total Number of Patients			102
Age (years)			
		Mean (St. dev)	63.8 (13.7)
		Median (range)	63.5 (30-90)
	Gender, n (%)		
		Female	39 (38.2%)
		Male	63 (61.8%)
	Tumor origin, n (%)		
		Primary	95 (93.18%)
		Recurrent	7 (6.9%)
	Differentiation, n (%)		
		Moderate	38 (37.3%)
		Poor	4 (3.9%)
		Well	60 (58.8%)
	Grade, n (%)		
		1	33 (32.4%)
		2	41 (40.2%)
		3	24 (23.5%)
		4	3 (2.9%)
		Unknown	1 (1.0%)

## Data Availability

Not applicable.

## Supplementary Materials

The following supporting information can be downloaded at: https://www.mdpi.com/article/10.3390/ijms232314624/s1.
