# Supplementary material for: Promoter Methylation of Cancer Stem Cell Surface Markers as an Epigenetic Biomarker for Prognosis of Oral Squamous Cell Carcinoma
Source: Int J Mol Sci. 2022 Nov 23;23(23):14624. doi: 10.3390/ijms232314624 (PMC9737199; doi:10.3390/ijms232314624)
Supplement: Supplementary file 1 [file ijms-23-14624-s001.zip › ijms-2021439-supplementary.pdf]

## Supplementary Figures and Table

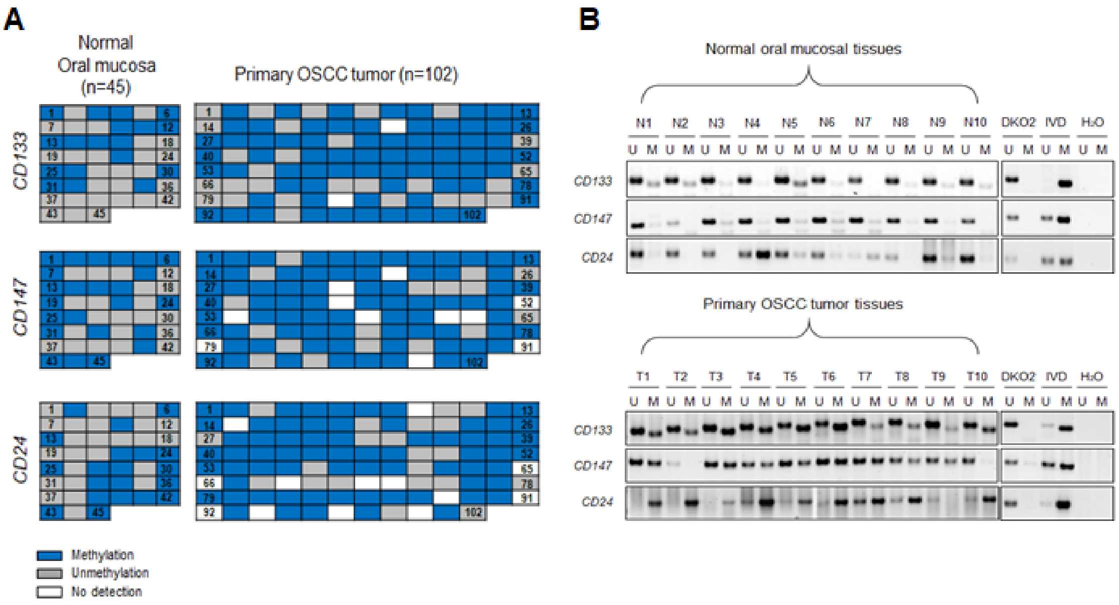


**Supplementary Figure S1 (related to Figure 2).** Summary of promoter DNA methylation analysis of *CD24, CD133,* and *CD147* genes in normal oral mucosal tissue (n=45) and primary OSCC tumor tissue samples (n=102). (A) Each square and numbers show a single patient sample. Blue and grey squares indicate methylated samples and unmethylated samples, respectively. (B) Representative MSP results in normal oral mucosa samples (n=10) and primary OSCC tumor samples (n=10). DNA methyltransferase 1 and 3b knockout HCT116 cells (DKO) were included as positive controls. PCR products recognize unmethylated (U) and methylated (M). DKO cells were used for the unmethylated control. IVD = in vitro methylated control; $H_2O$ = water control containing no DNA.

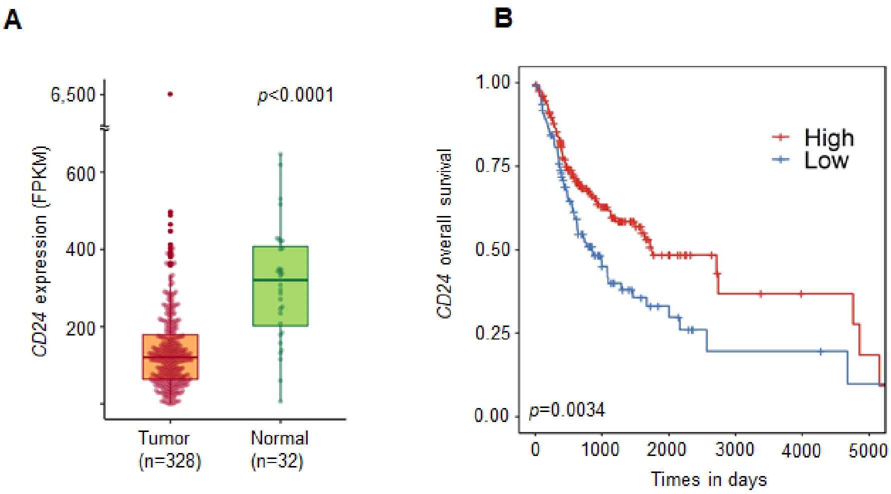


**Supplementary Figure S2.** Clinical implication of *CD24* gene using HNSCC TCGA database analysis. (A) The transcriptional expression pattern of CD24 gene between normal oral mucosa and primary OSCC tumors from HNSCC TCGA database (B) Kaplan-Meier curves

showing the effect of CD24 gene expression on overall survival among patients with OSCC (n=338) from HNSCC TCGA dataset. A probability of < 0.05 (log rank test *p < 0.05) was considered to represent a statistically significant difference. Clinical information of patients with OSCC (n=338) were divided into two groups according to CD24 gene expression status (expression high (red line), n=210; expression low (blue line), n=112).

**Supplementary Table S1.** Primer information for MSP, Bisulfite sequencing, and RT-PCR analyses in this study

| Genes | Targets | Primers | Sequences (5' - 3') |
|---|---|---|---|
| ***CD133**** | Unmethylation | CD133-F | TTATTATGGTGGTTTTATATTTAGGTTTTTGTTTG |
| | | CD133-R | ACTACAACCAAACCCTCAAACATAACA |
| | Methylation | CD133-F | GGCGGTTTTATATTTAGGTTTTCGTTC |
| | | CD133-R | CGAACCCTCGAACGTAACG |
| | RT-PCR | CD133-F | CCTGGGGCTGCTGTTTATTA |
| | | CD133-R | TACCTGGTGATTTGCCACAA |
| | BS-Seq | CD133-F | GTGAGTATGTTTAAGGAATTTTTT |
| | | CD133-R | CTAATCACAAATACCTCTCTC |
| ***CD147*** | Unmethylation | CD147-F | TGGGATGTAATTTTTAGAGTATATGG |
| | | CD147-R | AAATTTTAAATTTTAACCAACCAAA |
| | Methylation | CD147-F | GGGATGTAATTTTTAGAGTATACGG |
| | | CD147-R | AAATTTTAAATTTTAACCAACCGAA |
| | RT-PCR | CD147-F | CTCCTCACCTGCTCCTTGAA |
| | | CD147-R | TCTGACGACTTCACAGCCTT |
| | BS-Seq | CD147-F | ATTTAAGTGTTTTTTAAGTATTTTTATTTG |
| | | CD147-R | AAATATATTACCCAAATTTTATCTAAATTA |
| ***CD24*** | Unmethylation | CD24-F | TTGTAGTTTGTAGTGTTAGGTAGTGG |
| | | CD24-R | TTACTTTATTCTATAAACACCTCATA |
| | Methylation | CD24-F | TTTGTAGTTTGTAGCGTTAGGTAGC |
| | | CD24-R | TACTTTATTCTATAAACGCCTCGTA |
| | RT-PCR | CD24-F | AACTAATGCCACCACCAAGG |
| | | CD24-R | CCTGTTTTTCCTTGCCACAT |
| | BS-Seq | CD24-F | TTTGTAGTTTGTAGCGTTAGGTAGC |
| | | CD24-R | TACTTTATTCTATAAACGCCTCGTA |
| ***CD44***** | Unmethylation | CD44-F | TTTGTGTTTAGGGATTTTTTAGTTTTTTTT |
| | | CD44-R | CCAATCTACACCAAACTCAACAACACA |
| | Methylation | CD44-F | CGCGTTTAGGGATTTTTTAGTTTTTTTCG |
| | | CD44-R | TACGCCAAACTCAACGACACG |
| | RT-PCR | CD44-F | CCATCCCAGACGAAGACAGT |
| | | CD44-R | CATTCTGGAATTTGGGGTGT |

* indicate the primer information from previously published as below.
*Yi et al., Cancer Research 2008, 68, 8094-8103, doi:10.1158/0008-5472.CAN-07-6208.
** Yi JM. Genes & Genomics 2012, 34, 299-304, doi:10.1007/s13258-012-0038-4.
